# Fatigue-Induced Neuromuscular Performance Changes in Professional Male Volleyball Players

**DOI:** 10.3390/sports11060120

**Published:** 2023-06-16

**Authors:** Damjana V. Cabarkapa, Dimitrije Cabarkapa, Shay M. Whiting, Andrew C. Fry

**Affiliations:** Jayhawk Athletic Performance Laboratory—Wu Tsai Human Performance Alliance, Department of Health, Sport and Exercise Sciences, University of Kansas, Lawrence, KS 66045, USA; dcabarkapa98@gmail.com (D.V.C.); whitingsm99@gmail.com (S.M.W.); acfry@ku.edu (A.C.F.)

**Keywords:** training load, monitoring, team sports, force plate, rating of perceived exertion

## Abstract

The purpose of the present study was to assess pre-post practice changes in countermovement vertical jump (CVJ) force-time metrics and to determine the relationship between internal and external load variables within a cohort of professional male volleyball players. Ten elite athletes competing in one of the top professional European leagues participated in the present study. While standing on a uni-axial force plate, each athlete performed three CVJs immediately prior to the regular training session. Each athlete wore an inertial measurement unit (Vert^TM^) through an entire practice from which the following external load metrics were obtained: *Stress* (i.e., an algorithm-derived metric used to quantify the percentage of high-impact movements), *Jumps* (i.e., the total number of jumps performed during the practice session), and *Active Minutes* (i.e., the total amount of time performing dynamic movements). Immediately post-practice, each athlete completed another set of three CVJs and reported their subjective measure of internal load using a Rating of Perceived Exertion (RPE) scale (Borg CR-10). While no statistically significant differences were observed in any of the force-time metrics examined in the present study pre-post practice (e.g., eccentric and concentric peak and mean force and power, vertical jump height, contraction time, countermovement depth), our findings indicate a strong positive association between RPE and *Stress* (r = 0.713) and RPE and *Jumps* (r = 0.671). However, a weak non-statistically significant correlation was observed between RPE and *Active Minutes* (r = −0.038), indicating that internal load seems to be more dependent on the intensity rather than the duration of the training session for this sport.

## 1. Introduction

Volleyball is an intermittent team sport characterized by frequent high-intensity movements (e.g., jumping, change of direction) and short periods of rest [1,2,3,4,5]. It is comprised of the offensive and defensive phases during which players are required to perform a variety of sport-specific motions such as attacking, blocking, serving, and setting [6,7]. Over recent years, various testing modalities (e.g., force plates, accelerometers) have been implemented during volleyball practice sessions and/or games to help coaches, sports scientists, and strength and conditioning practitioners obtain a deeper insight into the training-related adaptations and neuromuscular fatigue, as well as the physiological workloads that athletes are exposed to during practice and competition [5,8].

Force plate technology has been commonly used for monitoring acute and chronic fatigue-induced neuromuscular changes in countermovement vertical jump (CVJ) performance [8,9]. Although some of the previous literature has only reported vertical jump height and performance metrics during the concentric phase of the jumping motion (e.g., peak power) [10,11], Merrigan et al. [8] indicated that the eccentric phase of the CVJ should not be overlooked. During the majority of the sport-specific movements, athletes are required to perform a combination of eccentric and concentric muscle actions, known as the stretch-shortening cycle [12]. Thus, in order to gain a better understanding of factors contributing to neuromuscular fatigue, both the eccentric and concentric phases should be assessed.

Previous research has focused on examining the effects of different fatiguing protocols on CVJ performance [11,13,14]. Gathercole et al. [11] found that peak force and eccentric function decreased immediately after a lower-body training session consisting of repeated stair climbs in elite snowboard athletes, while vertical jump height increased. Similarly, Cooper et al. [13] showed that acute fatigue (i.e., Bosco protocol) caused a decrease in vertical jump height, peak force, velocity, and power in recreationally active individuals. However, significant improvements in peak power, velocity, and displacement during CVJ were observed following a 12-week power training protocol, which could be attributed to the neuromuscular adaptations [14]. While the aforementioned findings identified several post-exercise fatigue-induced changes in CVJ performance, there is still a lack of scientific literature focused on examining the influence of volleyball-specific training activities on athletes’ state of fatigue and recovery.

With rapid technological developments over the recent decade, practitioners have been using different testing methods to quantify the workloads that athletes are exposed to during training and/or competition [5,15,16]. In a sport-setting, the workload has been previously defined by Gabbett et al. [17] as “the cumulative amount of stress placed on an individual during a training or competition, expressed as either external load (e.g., resistance lifted) or internal load (e.g., rating of perceived exertion - RPE).” It has been found that internal and external load variables tend to have a moderate to strong correlation [5,18]. Lima et al. [18] monitored professional male volleyball players over the course of 15 weeks and showed that a session RPE and the number of jumps had a moderate positive correlation (r = 0.49). Similarly, Vlantes and Readdy [5] found a strong positive correlation between RPE and player load (i.e., a sum of all accelerations across all axes during a movement; r = 0.73) and a moderate correlation between RPE and the number of jumps performed (r = 0.54) within a cohort National Collegiate Athletic Association (NCAA) Division-I female volleyball players. Moreover, the authors indicated that defensive specialists recorded the highest session RPE across 15 matches, while setters had the greatest player loads and the highest number of jumps [5]. Similar observations were made by Cabarkapa et al. [19], showing that setters during practice performed the highest number of jumps (~123) when compared to the rest of the playing positions (e.g., middle blockers, outside hitters, opposite hitters). Although no position-specific differences were observed in the *Stress* external load metric (i.e., an algorithm-derived variable that resembles the overall amount of stress placed on an athlete during practice sessions and/or game), all players seemed to experience medium to high levels of *Stress* throughout three consecutive training sessions [19].

Thus, considering the importance of workload monitoring for optimizing on-court performance, along with the lack of scientific literature focused on examining acute fatigue-induce changes in the neuromuscular performance of elite athletes, the purpose of the present study was to: a) assess pre-post changes in force-time metrics during both concentric and eccentric phases of CVJ and b) determine the relationship between internal and external load variables within a cohort of elite professional male volleyball players.

## 2. Materials and Methods

### 2.1. Participants

Ten professional male volleyball players (x ± SD; age = 21.4 ± 2.2 years; height = 197.3 ± 7.0 cm; body mass = 87.1 ± 7.3 kg; playing experience = 11.3 ± 2.4 years) volunteered to participate in the present study. The inclusion criteria involved athletes with >7 years of organized playing experience who were part of a professional volleyball team currently competing in one of the top European leagues (i.e., SuperLeague) at the time point of data collection. The athletes with musculoskeletal injuries that could possibly impair their on-court playing performance or athletes not cleared by their respective sports medicine staff to participate in team activities were excluded from participation in the present study. The testing procedures were approved by the University of Kansas Institutional Review Board (#00148619), and all athletes signed the informed consent form.

### 2.2. Testing Protocol

All testing protocols were performed during the mid-season competitive period, approximately 3–4 days post-last game. Upon arrival at the volleyball facility, all athletes were individually familiarized with the testing procedures and proceeded with performing a 15 min standardized team warm-up routine administered by their respective strength and conditioning coaches. Prior to the start of the regular practice session, each athlete stepped on a uni-axial force plate system (VALD, ForceDecks, Queensland, Australia) sampling at 1000 Hz and performed three maximal-effort CVJs with no arm-swing (i.e., hands on the hips during the entire movement). To minimize a possible influence of fatigue, each jump was separated by 10–15 s of passive rest [8]. The force plate system was zeroed/recalibrated between sets of three CVJs. Then, each athlete was provided with an individually assigned inertial measurement unit (IMU; Vert^TM^, Fort Lauderdale, FL, USA) to place around the waist (i.e., iliac crest) and secure with an elastic band. The IMU device was worn from the beginning to the end of the practice session, and the external load data was wirelessly collected at 1000 Hz via the Vert^TM^ Coach application installed on an iPad (Apple Inc., Cupertino, CA, USA). The practice session lasted approximately 90 min. It consisted of offensive and defensive drills (20 min), position-specific drills (20 min), and 6-on-6 competitive play (50 min). Immediately after the completion of the training session, athletes completed another set of CVJs following the aforementioned testing procedures. Also, the athletes’ subjective measure of the internal load was assessed post-practice via the Borg CR-10 scale for the Rating of Perceived Exertion (RPE) [20]. A score of “1” indicated minimal exertion, and a score of “10” indicated maximal exertion. Throughout CVJ testing procedures, athletes were verbally encouraged to give maximal effort and focus on pushing the ground as explosively as possible [21]. All data collection was performed by the same group of trained researchers and certified strength and conditioning specialists that perform similar testing procedures on a daily/weekly basis. 

### 2.3. Variables

Based on previous research, the following force-time metrics were obtained from the force plate due to demonstrating good reliability and practical applicability: braking phase duration and impulse, eccentric and concentric duration, peak velocity, mean and peak force and power, countermovement depth, contraction time, vertical jump height, and reactive strength index-modified (RSI-modified) [22,23,24]. A detailed description of the dependent variables examined in the present study can be found in the VALD user manual (https://valdperformance.com/forcedecks/, accessed on 6 June 2023) as well as previously published research reports [22,23,24].

The external load metrics obtained from the IMU device at the end of the training session were: *Stress* (i.e., an algorithm-derived metric used to quantify the percentage of high-impact movements through a training session; ≤15% low; 16–21% medium; ≥22% high), *Jumps* (i.e., the total number of jumps performed during a training session), and *Active Minutes* (i.e., the total amount of time performing dynamic movements) [19].

### 2.4. Statistical Analysis

Descriptive statistics means and standard deviations (x ± SD) were calculated for each dependent variable examined in the present study. Based on unpublished pilot data collected in our lab, a priori analysis indicated that the sample size (n = 10) was appropriate (d = 1.0; α = 0.05; β = 0.8). Shapiro–Wilk’s test corroborated that the assumption of normality was not violated. A paired-sample t-test was used to examine statistically significant (*p* < 0.05) pre-post changes in force-time metrics of interest. Based on the sample size (n < 20), Hedge’s *g* was used to calculate the measure of effect size (i.e., *g* = 0.2 small effect, *g* = 0.5 moderate effect, and *g* > 0.8 large effect) [25]. In addition, Pearson product-moment correlation coefficients were used to measure the strength of linear regressions between internal (i.e., RPE) and external load metrics (i.e., *Stress*, *Active Minutes*, *Jumps*) separately for each variable. Statistical significance was set a priori to *p* < 0.05. All statistical analyses were completed with SPSS (Version 26.0; IBM Corp., Armonk, NY, USA).

## 3. Results

**Figure 1:**
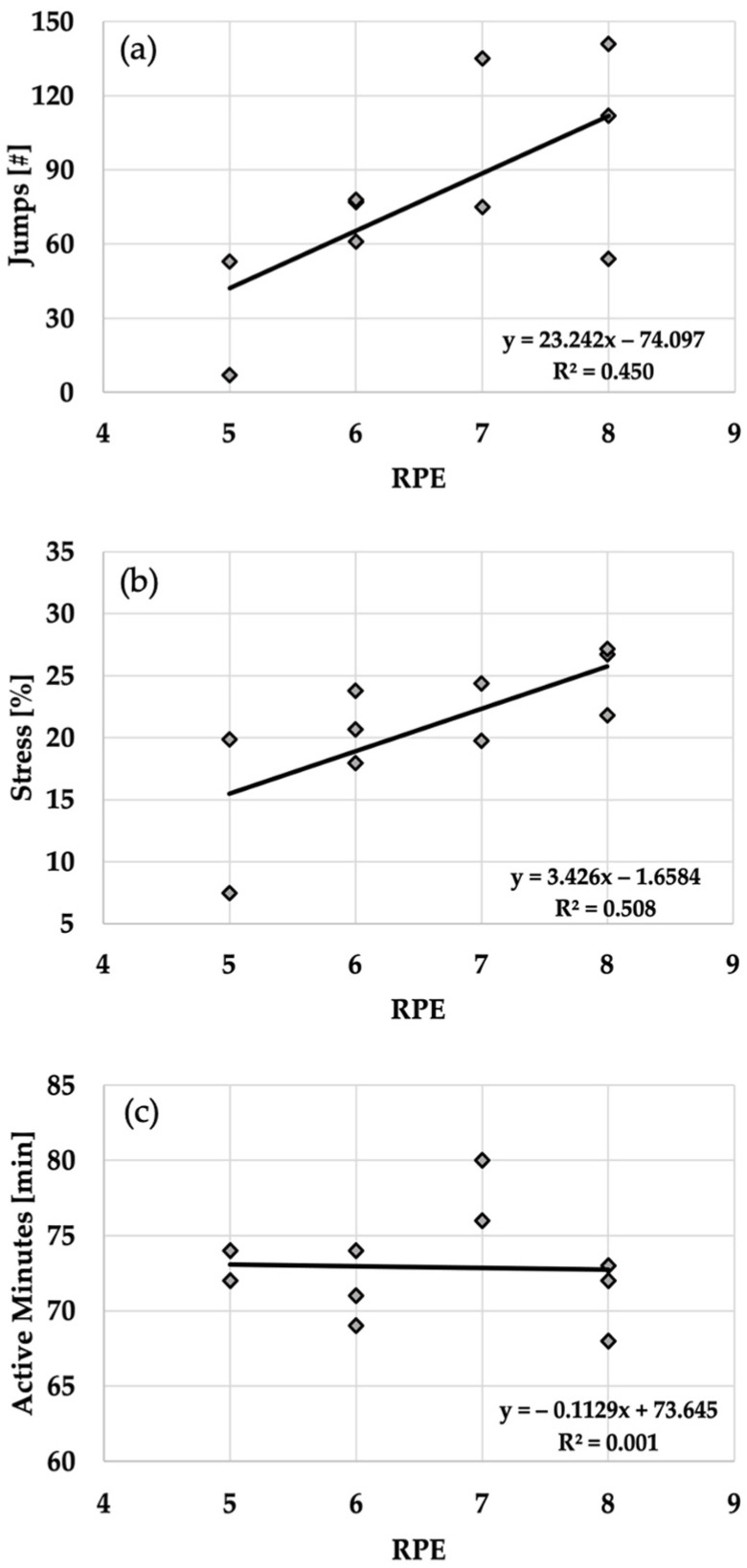
Relationship between subjective measure of internal load (rating of perceived exertion—RPE) and (**a**) *Jumps*, (**b**) *Stress*, and (**c**) *Active Minutes* external load metrics obtained from inertial measurement unit.

Descriptive statistics for each dependent variable examined in the present study are presented in Table 1 and Figure 1. No statistically significant differences in CVJ force-time metrics were observed pre-post practice (Table 1). All variables demonstrated trivial to small changes in magnitude (*g* = 0.000–0.310). On the other hand, RPE (6.60 ± 1.17) revealed a strong statistically significant correlation with *Stress* (20.95 ± 5.64%; r = 0.713, R^2^ = 0.508, *p* = 0.021) and *Jumps* (79.30 ± 40.69, r = 0.671, R^2^ = 0.450, *p* = 0.034) external load metrics obtained from the IMU device, while a weak non-significant negative correlation was observed between RPE and *Active Minutes* (72.90 ± 3.44 min; r = −0.038, R^2^ = 0.001, *p* = 0.916; Figure 1).

## 4. Discussion

To the best of our knowledge, this is the first study carried out in a non-laboratory setting that aimed to examine fatigue-induced changes in CVJ force-time metrics pre-post practice within a cohort of professional male volleyball players. Alongside CVJ assessment, athletes’ internal and external loads were quantified by using an RPE and wearable IMU device, respectively. While no statistically significant differences in force-time metrics were observed pre-post practice, our findings indicate the presence of a strong positive association between RPE and *Stress* (r = 0.713) and RPE and *Jumps* (r = 0.671). However, a weak non-statistically significant correlation was observed between RPE and *Active Minutes* (r = −0.038). 

Previous literature has reported mixed findings regarding fatigue-induced changes in CVJ force-time metrics [10,11,13,14,26,27]. When examining the effect of a lower-body muscular fatigue protocol (i.e., repeated stair climbs) on CVJ performance in elite snowboard cross athletes, Gathercole et al. [11] observed a notable decrease in peak concentric force and eccentric function (i.e., force at zero velocity and force-velocity area under the curve), as well as an increase in the jump time duration. Similar findings were noted by Cooper et al. [13], who found that the Bosco jump protocol significantly decreased peak concentric force and velocity, rate of velocity development, and impact force during the CVJ in recreationally active individuals. The aforementioned findings are contradictory to the results of the present study, where no significant changes in CVJ force-time variables were observed pre-post practice. These discrepancies may be mainly attributed to the differences in sport-specific physiological requirements (e.g., volleyball vs. snowboarding), the type, duration, and intensity of the training and/or testing protocols performed, as well as the athlete’s level of preparedness (e.g., recreational vs. professional). On the other hand, our results seem to be in agreement with an investigation done by Hoffman et al. [26] that focused on the assessment of anaerobic power performance during an NCAA Division-I intercollegiate American football game. The authors found that peak concentric force and power experienced a notable decrease post-second quarter when compared to the baseline levels (i.e., 10 min before the kickoff). However, the observed suppression in lower-body neuromuscular performance dissipated post-game, and both metrics displayed a tendency to return to the baseline levels [26]. In addition, it should be noted that the kinematic characteristics of the CVJ could have been another contributing factor to the previously mentioned differences. In a recently published study, Yu et al. [28] indicated that the running-induced fatigue protocol significantly altered lower-limb biomechanics, increasing the ankle and knee joints’ range of motion in the frontal plane during both the push-off and landing phases of the vertical jumping motion. Although kinematic characteristics such as joint angles were not assessed in the present investigation, the closest variable capable of depicting CVJ mechanics is the countermovement depth. Based on the trivial changes in the countermovement depth observed in the present study, we can assume that athletes’ jumping technique tended to remain unchanged pre-post practice sessions. Still, further research is warranted on this topic to determine if the discrepancies between previously mentioned research reports are primarily driven by neuromuscular fatigue, changes in jump mechanics, or both.

In addition to the CVJ testing on a portable force plate system, the present study quantified the internal and external training loads that volleyball players were exposed to during the practice session as well as the relationships between them. Our results revealed that the RPE scores reported by the players immediately after post-training sessions were strongly correlated with *Stress* and *Jumps* external load metrics (r = 0.713 and r = 0.671, respectively). This is in line with previous observations made by Vlantes and Readdy [5], who reported that player load (i.e., external load metric similar to the *Stress* variable) and the number of jumps quantified during fifteen NCAA Division-I women’s collegiate volleyball games had a high association with RPE (r = 0.73 and r = 0.54, respectively). In addition, the findings of a recently published investigation revealed that session RPE and the total number of jumps in professional male volleyball players competing at the Division-I level in Portugal demonstrated a moderate positive relationship (r = 0.49) [18]. Thus, we may conclude that RPE as a non-cost-prohibitive testing modality can be effectively used in a practical setting for the assessment of the athletes’ internal load. However, in order to appropriately interpret the RPE scores, it is important to consider the athletes’ levels of competition. Elite athletes may be more accustomed to sustaining higher levels of stress than athletes playing at lower levels of competition due to having greater training loads (i.e., dense practice schedule) and overall experience in their respective sports. For example, athletes in the present study who competed in one of the highest levels of the European league (e.g., SuperLeague) reported moderate levels of exertion (i.e., RPE = 6.6 ± 1.2) while sustaining a considerable amount of external load (i.e., *Stress* = 20.9 ± 5.6%). These observations are almost identical to the findings obtained by Svilar et al. [29], who found that elite male basketball players competing in the ACB League reported moderate levels of RPE, with an average of 6.6 ± 1.5, while experiencing substantial external loads (i.e., player load = 314.9 ± 90.0). Further, it should be noted that each volleyball-playing position seems to have unique physiological demands [5,19]. Although not examined in the present study, it has been previously shown that setters sustain the greatest amount of external load and perform the highest number of jumps during both practice sessions and games when compared to the other positions (e.g., outside hitters, opposite hitters, middle blockers) [5,19]. Interestingly, despite having the greatest amount of external load, setters did not report the highest session RPE scores [5]. Thus, further research is warranted on this topic to better understand the positional requirements in volleyball and if they induce different levels of acute neuromuscular fatigue. Lastly, our results revealed a weak non-statistically significant correlation between RPE and *Active Minutes* (r = −0.038), which implies that an increase in internal load (i.e., RPE) is more dependent on the training regimen design (i.e., intensity) than the overall session duration for these volleyball athletes. 

While the findings of the present investigation provide valuable information regarding training load monitoring and the impact of the volleyball practice session on acute neuromuscular fatigue, this study is not without limitations. The sample of athletes that participated in the present investigation was homogenous (e.g., elite male professional athletes) and limited in size. Hence, future research should focus on examining if our findings remain applicable to different levels of volleyball competition (e.g., collegiate) as well as if they are gender specific. In addition, all testing procedures were performed during a regular practice session. It is possible that these findings could have been altered if the testing protocol occurred during an official competitive match (e.g., audience, referees, presence of the opponent). 

## 5. Conclusions

In conclusion, the findings of the present study reveal that a regular practice session does not elicit significant changes in CVJ force-time metrics pre-post practice in elite male volleyball players and that internal load seems to be more dependent on the intensity (i.e., *Stress*, *Jumps*) rather than the duration of the training session (i.e., *Active Minutes*). These results suggest that quantifying the fatigue-induced neuromuscular performance changes should not be based on a single test metric. Using the aforementioned testing modalities (e.g., CVJ assessment, external and internal load monitoring) together has greater practical application as it can help coaches, sports scientists, and strength and conditioning practitioners to obtain better insight into on-court performance demands that athletes are exposed to during regular practice sessions and may aid in optimizing athletes’ longitudinal development.

## Figures and Tables

**Table 1:** Pre-post practice changes in force-time metrics. A downward arrow (↓) represents a % change decrease, an upward arrow (↑) represents a % change increase, and a dash (–) represents no change pre- to post-practice.

Variable [Unit]	Pre-Practice	Post-Practice	*p*-Value	Effect Size	% Change
Eccentric phase					
Braking phase duration [s]	0.36 ± 0.11	0.35 ± 0.08	0.590	0.104	↓ 2.78
Eccentric braking impulse [N·s]	59.19 ± 18.64	59.17 ± 12.39	0.997	0.001	↓ 0.03
Eccentric duration [s]	0.66 ± 0.11	0.63 ± 0.13	0.324	0.249	↓ 4.55
Eccentric peak velocity [m·s^−1^]	−1.18 ± 0.24	−1.18 ± 0.20	0.989	0.000	–
Eccentric peak force [N]	1918.50 ± 253.43	1912.00 ± 289.91	0.865	0.024	↓ 0.34
Eccentric mean force [N]	843.00 ± 67.25	857.60 ± 73.88	0.409	0.207	↑ 1.73
Eccentric peak power [W]	1321.80 ± 382.81	1384.90 ± 375.27	0.529	0.166	↑ 4.77
Eccentric mean power [W]	478.90 ± 73.97	493.50 ± 85.19	0.527	0.183	↑ 3.05
Concentric phase					
Concentric impulse [N·s]	242.94 ± 22.68	245.57 ± 25.06	0.263	0.110	↑ 1.08
Concentric duration [s]	0.30 ± 0.04	0.30 ± 0.04	0.711	0.000	–
Concentric peak velocity [m·s^−1^]	2.96 ± 0.31	2.93 ± 0.21	0.600	0.113	↓ 1.01
Concentric peak force [N]	2010.80 ± 178.78	2051.80 ± 179.49	0.108	0.229	↑ 2.04
Concentric mean force [N]	1663.80 ± 178.60	1694.90 ± 187.64	0.263	0.169	↑ 1.87
Concentric peak power [W]	4817.80 ± 638.22	4878.20 ± 511.37	0.430	0.104	↑ 1.25
Concentric mean power [W]	2558.20 ± 477.12	2554.30 ± 406.70	0.947	0.009	↓ 0.15
Other					
Contraction time [s]	0.95 ± 0.12	0.94 ± 0.18	0.772	0.065	↓ 1.05
Vertical jump height [cm]	41.88 ± 9.82	40.73 ± 6.25	0.611	0.139	↓ 2.75
RSI-modified [m·s^−1^]	0.43 ± 0.08	0.46 ± 0.11	0.145	0.312	↑ 6.98
Countermovement depth [cm]	−37.18 ± 7.55	−36.87 ± 6.45	0.872	0.044	↓ 0.83

Note: RSI-modified = reactive strength index modified.

## Data Availability

The data presented in this study are available on request from the corresponding author.
